# Comprehensive study of semi-supervised learning for DNA methylation-based supervised classification of central nervous system tumors

**DOI:** 10.1186/s12859-022-04764-1

**Published:** 2022-06-08

**Authors:** Quynh T. Tran, Md Zahangir Alom, Brent A. Orr

**Affiliations:** Department of Pathology, St. Jude Children’s Research Hospital, 262 Danny Thomas Place, MS 250, Memphis, TN 38105-3678 USA

**Keywords:** Semi-supervised learning, Neural network, Artificial intelligence, Supervised classifiers, DNA-methylation, Central nervous system tumor, Machine learning, Random forest

## Abstract

**Background:**

Precision medicine for cancer treatment relies on an accurate pathological diagnosis. The number of known tumor classes has increased rapidly, and reliance on traditional methods of histopathologic classification alone has become unfeasible. To help reduce variability, validation costs, and standardize the histopathological diagnostic process, supervised machine learning models using DNA-methylation data have been developed for tumor classification. These methods require large labeled training data sets to obtain clinically acceptable classification accuracy. While there is abundant unlabeled epigenetic data across multiple databases, labeling pathology data for machine learning models is time-consuming and resource-intensive, especially for rare tumor types. Semi-supervised learning (SSL) approaches have been used to maximize the utility of labeled and unlabeled data for classification tasks and are effectively applied in genomics. SSL methods have not yet been explored with epigenetic data nor demonstrated beneficial to central nervous system (CNS) tumor classification.

**Results:**

This paper explores the application of semi-supervised machine learning on methylation data to improve the accuracy of supervised learning models in classifying CNS tumors. We comprehensively evaluated 11 SSL methods and developed a novel combination approach that included a self-training with editing using support vector machine (SETRED-SVM) model and an L2-penalized, multinomial logistic regression model to obtain high confidence labels from a few labeled instances. Results across eight random forest and neural net models show that the pseudo-labels derived from our SSL method can significantly increase prediction accuracy for 82 CNS tumors and 9 normal controls.

**Conclusions:**

The proposed combination of semi-supervised technique and multinomial logistic regression holds the potential to leverage the abundant publicly available unlabeled methylation data effectively. Such an approach is highly beneficial in providing additional training examples, especially for scarce tumor types, to boost the prediction accuracy of supervised models.

**Supplementary Information:**

The online version contains supplementary material available at 10.1186/s12859-022-04764-1.

## Background

Artificial intelligent (AI) technologies have been widely adopted in the diagnostic process of various biomedical disciplines [1–4]. Furthermore, with the advent of high-throughput technologies such as microarrays and nucleic acid sequencers, the use of machine learning and deep learning has also become increasingly indispensable in the field of cancer genomics [5–7]. The introduction of these advanced computational methods has provided many opportunities to improve health care and increase the precision of oncologic diagnosis.

A key challenge in medical science is the precise classification of diseases and the development of optimal therapies. This is particularly more challenging in classifying brain tumors due to the developmental complexity of the brain. The World Health Organization has defined 82 central nervous system (CNS) tumor classes, encompassing a broad spectrum from benign neoplasms, which can be treated by surgery alone, to malignant tumors that respond poorly even with aggression adjuvant therapy. With the advancement in AI and the abundance of genomic and epigenomic data, methylation-based classification of human tumors has emerged as an essential diagnostic tool in the clinical laboratory. Supervised models have been implemented to assist in diagnosing CNS tumors and sarcomas [8, 9].

These initially deployed models are clinically useful but have inherent limitations. Constructing optimal supervised models for methylation-based classification in the clinical environment is dependent on having a comprehensive set of labeled “gold standard” data for training and validation. Unfortunately, the current reference sets are not entirely complete, yielding a significant proportion of unclassifiable tumors [8]. Furthermore, the reference cohorts suffer from a considerable class imbalance due to the lack of sufficient examples of rare tumor types to train supervised classification models, thus, degrading model performance.

To fully leverage methylation profiling and machine learning for tumor classification, models should be improved over time by augmenting the training cohorts with additional labeled reference examples of rare tumors and relabeling samples after additional molecular substructures have been identified within known tumor types. In addition, with the vast publically available methylation profiling data, model updates would benefit from combining well-characterized data with relevant tumor profiles acquired from large public repositories.

Obtaining additional labeled training data for improving CNS tumor classifiers can be challenging. Current “gold standard” approaches to sample labeling for methylation cohorts include a histomorphologic assessment by expert pathologists, orthogonal molecular testing, and unsupervised methods such as dimensionality reduction or cluster analysis. However, establishing a ground truth methylation class is difficult for a subset of tumors because they lack defining gene abnormalities or copy number changes. Additionally, closely related molecular subgroups within tumor types can be challenging to distinguish unbiasedly. Therefore, the cost of time, effort, and additional testing make this degree of rigor in labeling infeasible, particularly when applied to large cohorts.

Semi-supervised learning (SSL), an intermediate approach between unsupervised (with no labeled training data) and supervised (with only labeled training data) learning, is often used when labeling data is not feasible or requires substantial resources [10, 11]. Depending on the objectives, SSL can be divided into classification [12], regression [13], or clustering [14]. In this study, our objective is the former, which focuses on enhancing supervised classification by minimizing errors in the labeled examples. There are two different learning settings in semi-supervised classification: transductive and inductive learning. Transductive learning predicts the labels of the unlabeled examples provided during the training phase. On the other hand, inductive learning predicts the labels of unseen data using the labeled and unlabeled data provided during the training phase [12].

Semi-supervised learning methods have shown great success in areas such as image recognition and natural language processing [15–19], and it has been applied to a diverse set of problems in biomedical science including image classification [20–24] and medical language processing [25–28]. These methods have been applied to classification tasks using image and natural language data, which relies on spatial and semantic structure, i.e. the spatial correlations between pixels in images and sequential correlations between words in the text. By contrast, healthcare and genomic problems primarily involve high-dimensional tabular data in which the inherent structures among features are unknown and vary across different data sets. Some examples of semisupervised learning applications in genomic medicine include gene finding, miRNA discovery, predicting gene regulatory networks, and survival modeling [12, 29–32]. Although SSL has shown to be effective in the genomic field, it has not been explored with epigenetic or methylation data from human tumors. Rare applications to brain tumor classification have been limited to deriving tumor classes from radiologic images [33].

In this study, and in the context of methylation-based CNS classification, we present the first application utilizing widely used semi-supervised learning algorithms known as “self-training” [34–36] and “co-training” [11, 37] to assign CNS tumors to 91 methylation subclasses. Our objective focuses on determining how the performance of supervised AI models used for CNS tumor classification is affected by the number of “labeled” individuals with known methylation classes and “unlabeled” individuals with SSL labels. This study demonstrates the efficient enhancement of CNS tumor prediction accuracy through the inclusion of patients without histopathological diagnosis labeled via semi-supervised learning methods. This approach is likely to be more broadly applicable to other types of cancer and biomedical data.

## Results

### Cohort characteristics

As an initial evaluation of SSL on methylation data, we used a cohort of previously published training data from a comprehensive brain tumor classification model (GSE90496 [8]). We applied five different training functions with four different base learners (Additional file 1: Table S1). This cohort has several characteristics that make it ideal for this task. The cohort includes 2801 labeled tumors comprising 75 methylation families (MCF) from tumor and standard brain samples, representing 82 distinct CNS tumor methylation subclasses (MC) and nine control groups. The subclasses represent closely related molecular groups within individual tumor types, and a comparison of the family and subclass labeling performance is a good surrogate for tumor relabeling. We used samples from GSE109379 for inductive learning. GSE109379 is a prospective cohort described in [8] consisting of 1,104 patients with given diagnostic categories comprising 64 different histopathological entities and pediatric cancers.

### Semi-supervised models for evaluation

We evaluated 11 semi-supervised learning models (Additional file 1: Table S1) using five different training techniques, i.e., self-training (SELFT [36]), self-training with editing (SETRED [34]), self-training nearest-neighbor rule using cut edges (SNNRCE [35]), tri-training (TRITRAIN [11]), and democratic co-learning (DEMO [37]). Each training function used either of these supervised classifiers as the base learner: one nearest neighbor (oneNN), decision tree C5.0, and support vector machine (SVM). For SNNRCE, oneNN was the fixed built-in learner. For DEMO, all three base learners were used. The learner parameters for SVM were C-classification and radial kernel. Euclidian distances were used for SSL with oneNN base learner.

### SSL models performance on tumor family classification

**Fig. 1:**
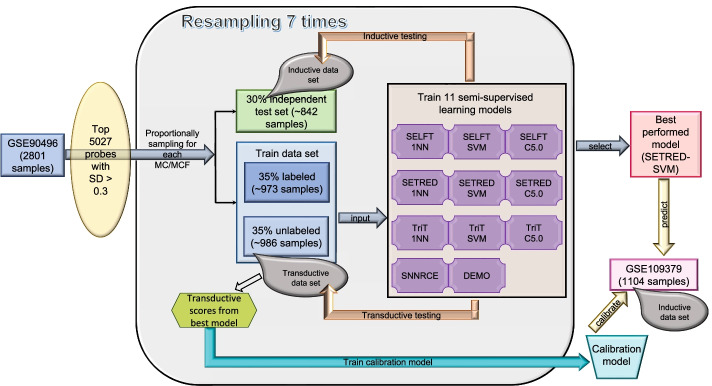
Training and testing scheme for the 11 evaluated SSL models. After preprocessing the GSE90496 methylation data, probes with a standard deviation greater than 0.3 across all 2,801 samples were selected as features used in the 11 SSL models. Thirty percent of the samples were kept aside as an inductive testing data set to independently evaluate the performance of each SSL model. The remaining 70% of the data was used as training sets. The training data were proportionally partitioned into a labeled and an unlabeled set. Specifically, 50% (of the 70%) training data were used as labeled examples, while the remaining 50% (of the 70%) data were used as unlabeled examples or as a transductive test set. The partitioning process was bootstrapped seven times. Each molecular methylation group was proportionally selected for every bootstrap to ensure that the class distributions were similar to the original class distributions

The train and test data for SSL models are depicted in Fig. 1. In our study, the performance of each model was summarized and evaluated based on average accuracy, specificity, precision, and recall after predicting 75 MCFs for 842 (30% of 2801 samples) inductive and 986 transductive samples.

**Fig. 2:**
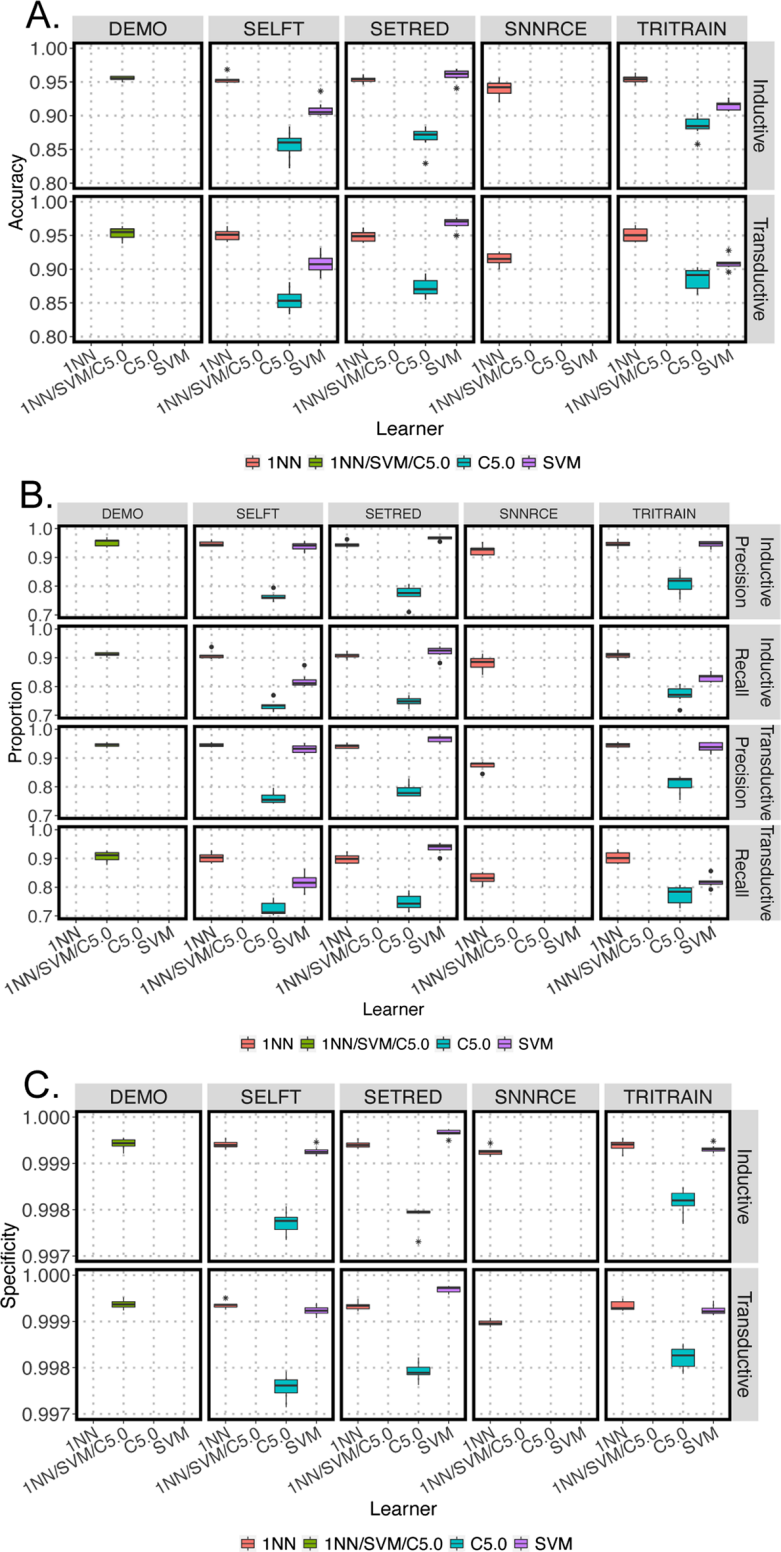
Box plots summarizing SSL performance of 11 SSL models for predicting 75 methylation class families (MCF) of inductive and transductive testing data. **A** Accuracy; **B** Precision and recall; **C** Specificity across all models. Box plots showing the results of all 7 bootstraps. Asterisks represent the outliers. 1NN–pink; combine of 1NN/SVM/C5.0–green; C5.0–cyan; and SVM–purple

All models performed well on transductive and inductive data sets at the family classification level with an accuracy above 0.84 (Fig. 2A). Training functions with the C5.0 decision tree as the base learner had the lowest average accuracy (< 0.87) (Fig. 2A) as well as precision (< 0.77) and recall (< 0.73) (Fig. 2B). Those with 1NN learners had consistent accuracies above 0.93 (Fig. 2A), while their precision and recall were around 0.92 and 0.87, respectively (Fig. 2B). Among the 11 models, SETRED with SVM base learner performed the best with mean accuracy above 0.95 (Fig. 2A), precision greater than 0.96, and recall above 0.91 (Fig. 2B). In addition, all models had very high specificity (above 0.99), suggesting that SSL could identify true negative at a high rate (Fig. 2C).

### SSL models performance on tumor subclass classification

**Fig. 3:**
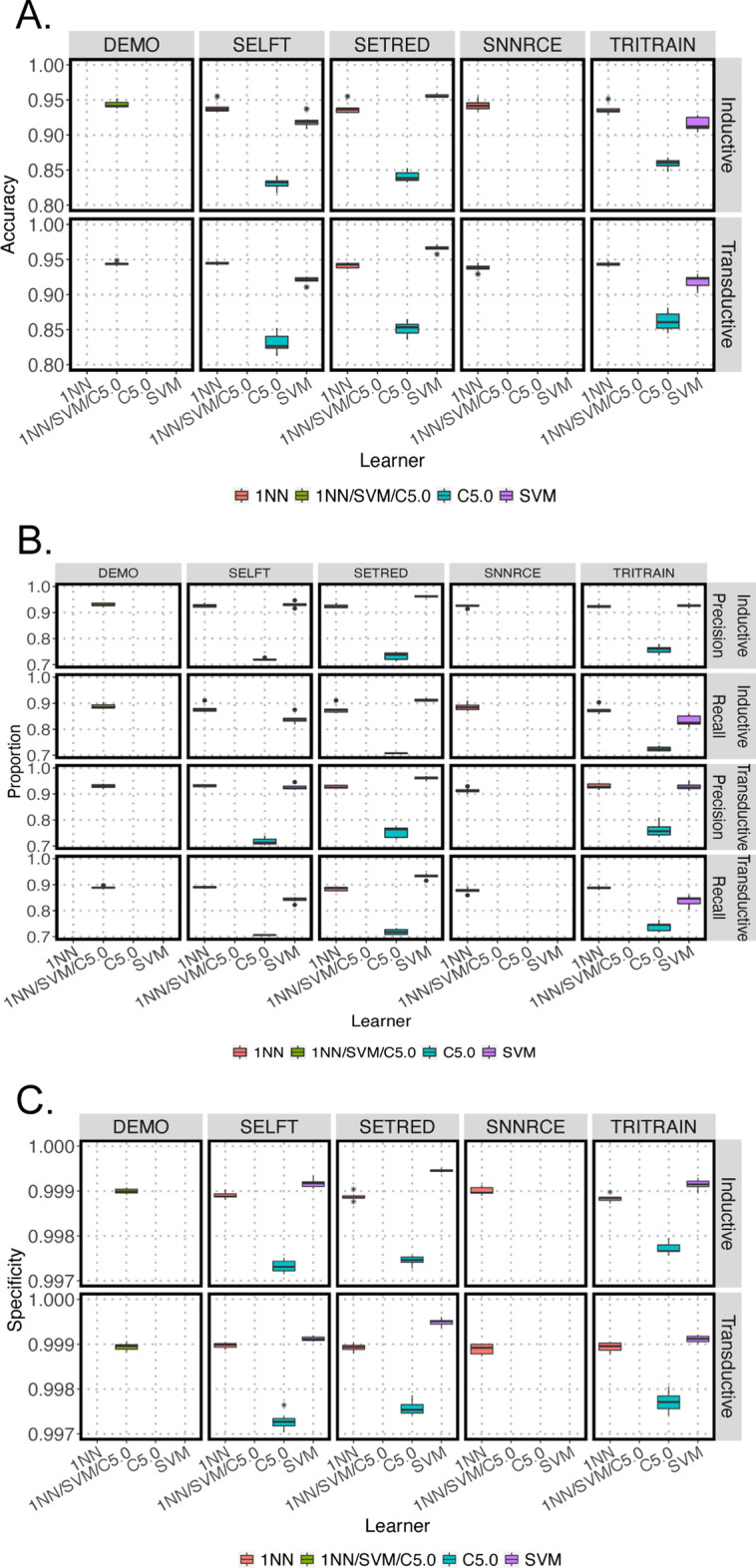
Box plots summarizing SSL performance of 11 SSL models for predicting 91 methylation subclasses (MC) of inductive and transductive testing data. **A** Accuracy; **B** Precision and recall; **C** Specificity across all models. Box plots showing the results of all 7 bootstraps. Asterisks represent the outliers. 1NN–pink; combine of 1NN/SVM/C5.0–green; C5.0–cyan; and SVM–purple

The performance of the 11 SSL models was also assessed based on their prediction of 91 methylation subclasses (MC). Figure 3 shows the prediction of the inductive and transductive data sets for each training function combined with different supervised-based learners. Similar to the methylation family classification, training function with 1NN and SVM (accuracy > 91%, precision > 91%, and recall > 83%) performed better than those with C5.0 decision tree (accuracy ranges from 83 to 86%, precision ranges from 70 to 76%, and recall ranges from 66 to 84%) (Fig. 3A and 3B). Among the 11 SSL models, SETRED with SVM base learner performed the best with accuracy ~ 97%, precision > 96%, and recall > 91% for inductive and transductive testing (Fig. 3A, B). All models had specificity at around 99% (Fig. 3C).

### Obtaining high confidence scores from the best SSL model

**Fig. 4:**
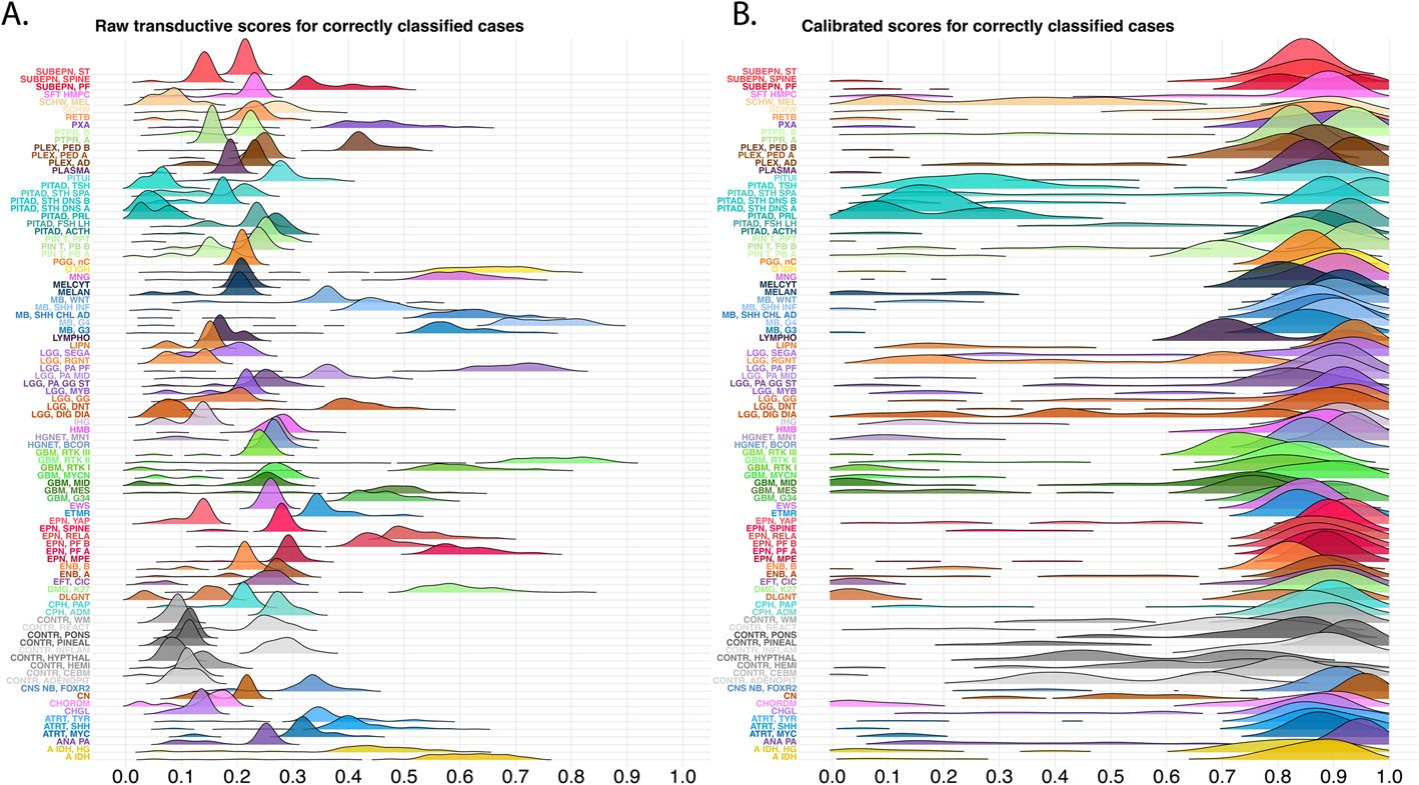
Distributions of SETRED-SVM predicted scores for 91 methylation classes. **A** Before calibration **B** After calibration for correctly classifiable (true positive) cases

After evaluating 11 SSL models, SETRED-SVM yielded the highest accuracy, precision, and recall and was chosen as an application model for further analysis. SETRED-SVM also produced the biggest AUC for methylation class and family prediction (AUC = 0.73 and 0.94, respectively) (Additional file 1: Fig. S1A-B). This model assigned a predicted score to each of the 91 classes for each sample, resulting in an aggregated raw score (Fig. 4A). To obtain class probability estimates that can measure the confidence in the class assignment, we fitted a multinomial logistic regression calibration model to the raw scores. This calibration process produced calibrated scores that allowed us to compare between classes despite differences in raw score distributions (Fig. 4B).

**Fig. 5:**
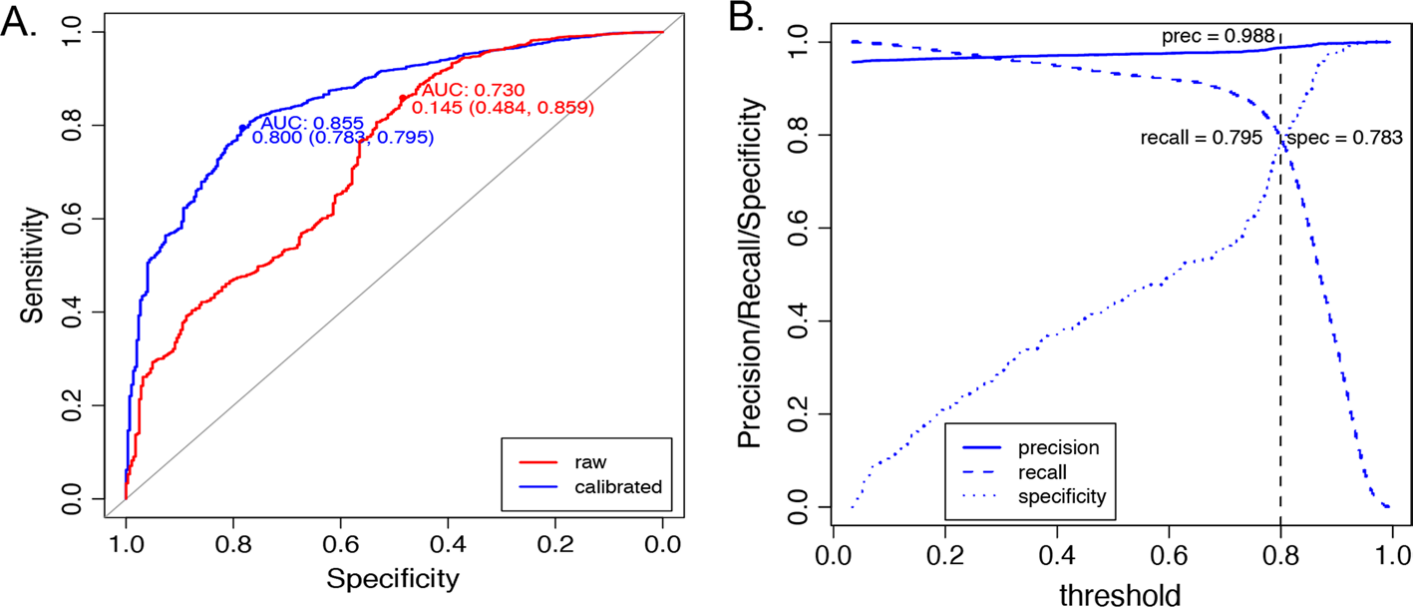
ROC and threshold analysis of SETRED-SVM model for classifiable and non-classifiable methylation subclass (MC) cases. **A** ROC analysis of maximal raw (red) and maximal calibrated (blue) predicted scores with the area under the curve (AUC) and the corresponding specificity and sensitivity that maximized the Youden Index. **B** Specificity, sensitivity (recall), and precision at different thresholds using calibrated scores. The black vertical line represents the suggested threshold (≥ 0.8) at which we had the best balance between sensitivity and specificity using calibrated scores

Receiver operating characteristic (ROC) curve analysis of the maximum raw and calibrated scores demonstrated that calibration improved the area under the curve (AUC) from 0.73 to 0.855 for predicting 91 methylation classes (Fig. 5A). To obtain a cutoff score with high confidence for predicting a matching class, we performed a threshold analysis that utilized maximization of the Youden Index (balanced between sensitivity and specificity) [38]. The analysis suggested a threshold of 0.8 for MC calibrated scores with a specificity of 78.3%, sensitivity of 79.5%, and precision of 98.8% (Fig. 5B). ROC analysis of SETRED-SVM and the other models using raw and calibrated scores are shown in Additional file 1: Fig. S1A-B. The specificity and sensitivity of SETRED-SVM for predicting methylation family at threshold 0.8 were 95.9% and 79.8%, respectively (Additional file 1: Fig. S1C).

### Effect of supervised classification model performance after augmenting training data with semi-supervised labeled data

**Fig. 6:**
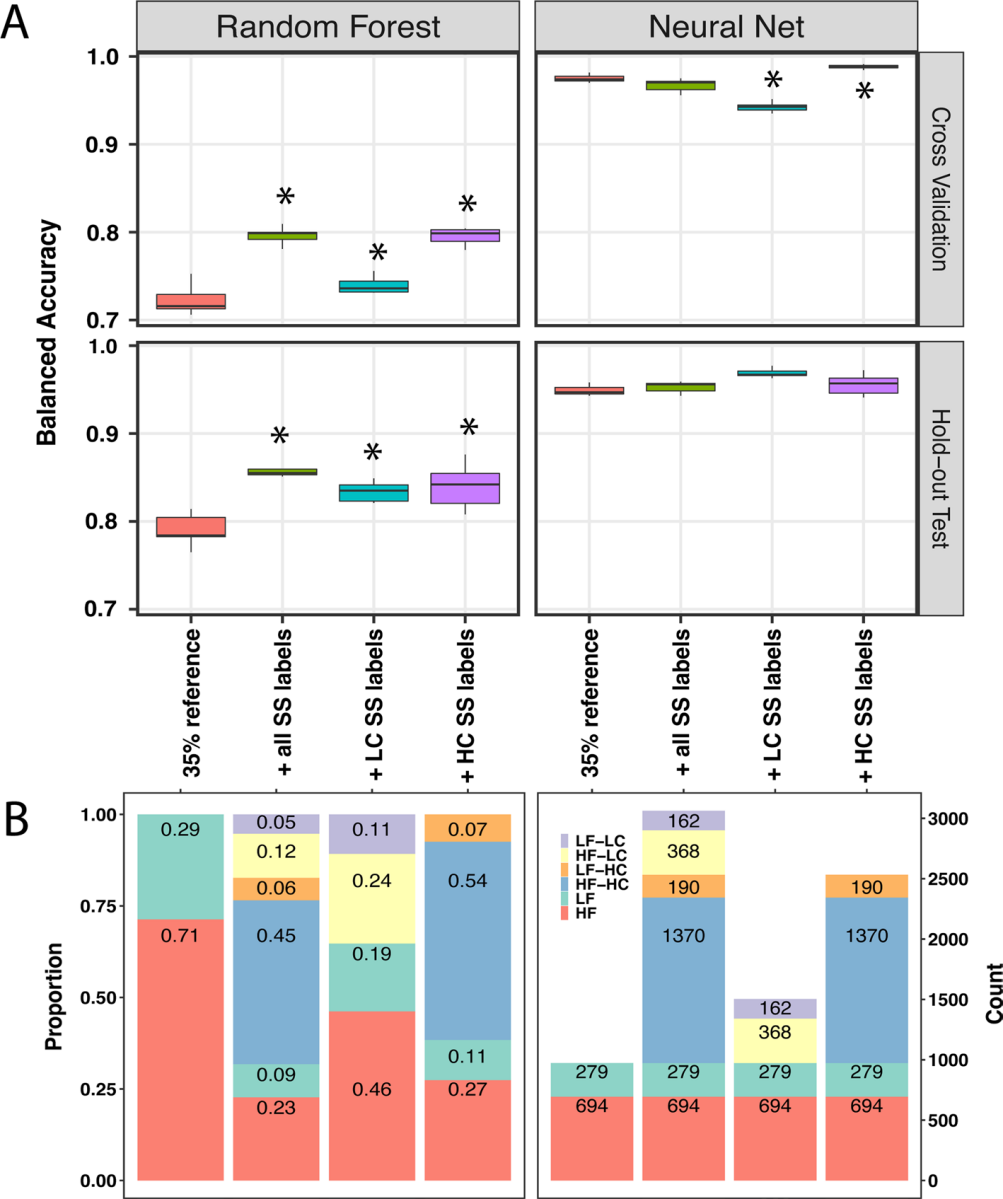
Prediction performance of random forest (RF) and neural net (NN) classifiers at family level when trained with different combination of reference samples and semi-supervised (SS) predicted labeled samples. **A** Balanced accuracy of RF and NN. **B** Proportion (left panel) and count (right panel) of high (≥ 10 samples, red) and low (< 10 samples, green) frequency referent labels, high confident (HC) SS labels with high frequency (blue) and low frequency (orange) families, and low confident (LC) SS labels in high frequency (yellow) and low frequency families (purple). Asterisks indicate statistically significant difference performed by Tukey Honest Significant Difference test at 0.05 alpha level

**Fig. 7:**
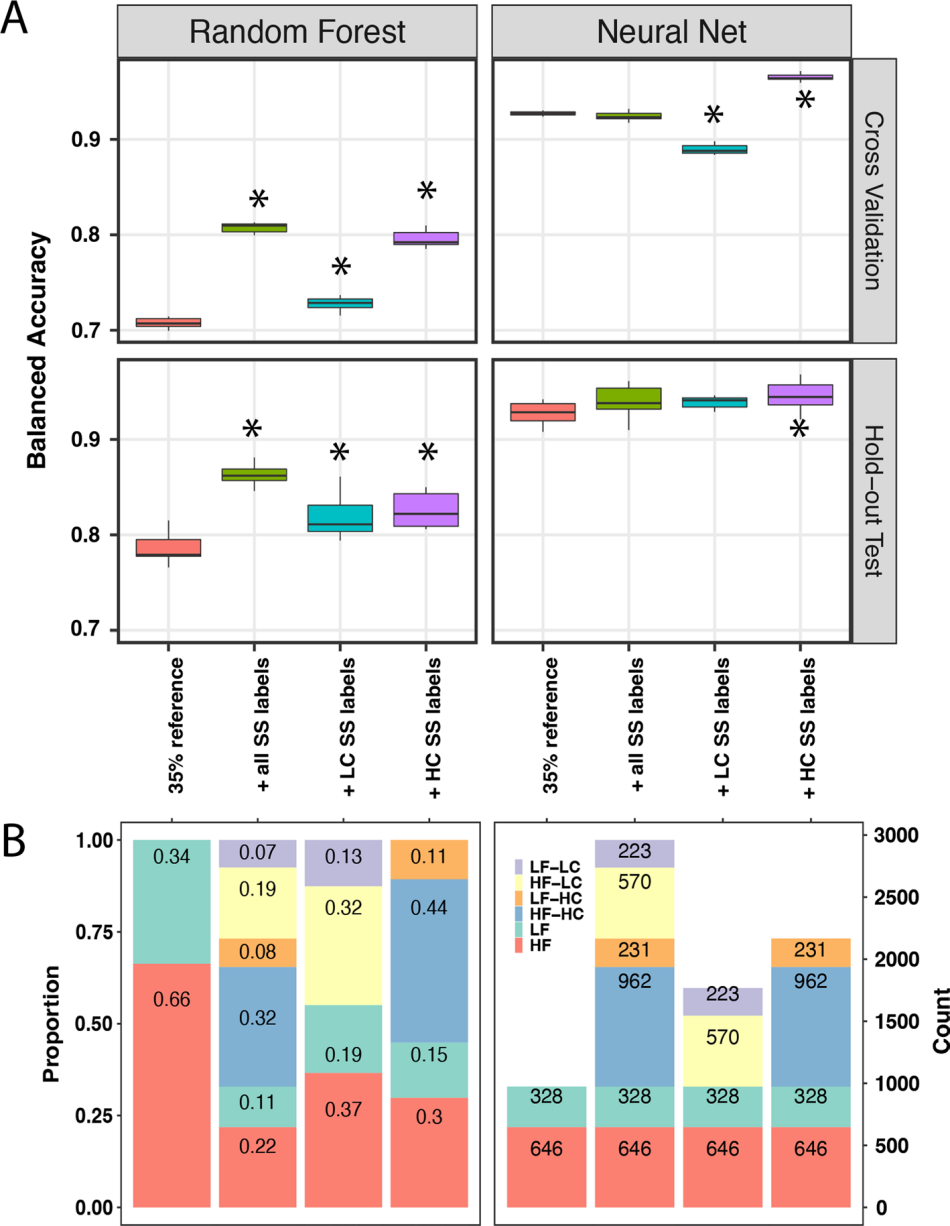
Prediction performance of random forest (RF) and neural net (NN) classifiers at sub-class level when trained with different combination of reference samples and semi-supervised (SS) predicted labeled samples. **A** Balanced accuracy of RF and NN. **B** Proportion (left panel) and count (right panel) of high (≥ 10 samples, red) and low (< 10 samples; green) frequency referent labels, high confident (calibrated SSL scores ≥ 0.8, HC) SS labels with high frequency (blue) and low frequency (orange) subclasses, and low confident (calibrated SSL scores < 0.8, LC) SS labels in high frequency (yellow) and low frequency (purple) subclasses. Asterisks indicate statistically significant difference performed by Tukey Honest Significant Difference test at 0.05 alpha level

To demonstrate the utility of our labeling method, we tested whether adding samples labeled using SSL would improve the performance of supervised random forest (RF) and neural net (NN) classification models. First, we built baseline models with 35% data from GSE90496. Then, we trained the baseline models with additional SSL pseudo-labels from the remaining 35% GSE90496 and the 1104 GSE109379 samples (Additional file 1: Fig. S2). Compared to the baseline model, all RF models showed a statistically significant increase in balanced accuracy (up to 7%), while the NN models showed a significant increased in balanced accuracy (0.7%) when only high confidence MCF labels were included (Fig. 6A). Overall, the NN model yielded a much higher balanced accuracy (92.9% and 97.5%) compared to the RF classifier (70.9% and 72.3%) at predicting methylation subclass and family, respectively (Fig. 6 and 7). Unlike the RF classifier, the performance of the NN on the hold-out test set appeared to be very robust (Fig. 6A and 7A). The RF prediction accuracy on the hold-out test set closely resembled its cross-validation performance. In contrast, the accuracy of the NN appeared to be unchanged regardless of its cross-validation performance for both subclass and family levels (Fig. 6A and 7A).

To further understand how the quality of semi-supervised labels affects the supervised classifiers, we have defined labels predicted by SETRED-SVM with calibrated scores above the thresholds as high confident (HC) while the remaining would be considered as low confident (LC) SS labels. The numbers and their corresponding proportions to the referent labeled samples were categorized into low frequency (< 10 samples per group, LF-green bars), high frequency (^3^ 10 samples per group, HF-red bars), as shown in Fig. 6B and 7B. We observed that adding more data to the original training data set showed a statistically significant improvement in the performance of the RF classifier (Fig. 6A and 7A). NN performance showed statistically significant improvement in the presence of high confident SS labels only, while its accuracy significantly decreased in the addition of only low confident data. This suggests that NN was more sensitive to the data quality than the RF classifier. In conclusion, semi-supervised labels improved the prediction of both RF and NN classifiers. The NN classifier had a higher accuracy prediction; however, its performance was more sensitive to low confident labels than the RF classifier.

## Discussion and conclusions

This study demonstrates that semi-supervised (SS) learning is a valuable and efficient approach to label or relabel large cohorts of unclassified samples using their DNA-methylation profiles. Further, we show that the pseudo-labels from an SSL model can effectively be utilized during training to improve the performance of supervised classification models. This approach substantially reduces the subjectivity and effort from domain experts associated with class label generation.

Among the 11 SSL models (Additional file 1: Table S1) evaluated, SETRED-SVM, a data editing method using support vector machine as the base learner, outperformed other models for labeling methylation family or subclass. SETRED utilizes an active-learning-like technique to identify and remove mislabeled examples from the self-labeled data and has been demonstrated to be robust to noise in self-labeled data [34]. While this is the first instance of SSL being applied to methylation array data, other investigators have also found SETRED to perform well in other biomedical SSL tasks such as biomedical image classification [39].

When using SSL methods to label data, one concern is the potential to spuriously label out-of-distribution samples with one of the provided seed labels [40]. Implementation of SSL for clinical classification tasks likely will require concomitant sample distribution analysis. In our instance, we calibrated the SETRED-SVM predictive scores using a multinomial logistic calibration model. The logistic model produced probability estimates that could be used to assess the confidence in class assignment. This approach was previously adapted to calibrate the RF classification scores for methylation-based brain tumor classification [8]. We showed that this step is critical in creating a high-quality data set for subsequent supervised classifier training. Receiver operating characteristic (ROC) curve analysis of SETRED-SVM maximum raw and calibrated scores demonstrated that calibration improved the area under the curve (AUC) from 0.73 to 0.855, respectively (Fig. 5A). The RF and NN classifiers showed significant degradation when low-confidence samples were utilized for training, even with a substantial increase in overall samples (Fig. 6 and 7).

One problem with the chosen method of distribution analysis was it yielded relatively low scores for tumors with very low representation (< 10 samples) among the seed labels. For instance, tumor classes such as pituitary adenoma, prolactin (PITAD, PRL) and low-grade glioma, desmoplastic infantile astrocytoma/ganglioglioma (LGG, DIG/DIA), which had representation from only eight tumors per methylation class, each failed to produce high-confidence pseudo-labeled tumors (Additional file 1: Figs. S3 and S4). We suspect that polynomial regression models may show better performance in determining the class confidence from under-represented tumor classes because they are more suitable for high-dimensional data [41] and provide plausible results for small sample size data (< 8) [42].

In this study, we evaluated 11 classical SSL models and found that they are highly effective for labeling CNS tumors using DNA-methylation profiles. Our findings prove that our approach is useful for our task; however, as our implementation utilized relatively simple models, future studies could explore whether more recent and state-of-the-art SSL approaches offer additional advantages. For instance, the semi-supervised classification with extensive knowledge exploitation (SSC-EKE) [43] and a systematic self- and semi-supervised learning framework, value imputation and mask estimation (VIME) [44] have demonstrated exemplary performance when applied to alternative tasks. SSC-EKE can minimize the empirical risk and control the model smoothness by extensive use of the knowledge embedded in the entire training data, regardless of the label availability, to construct the unbiased approximation of the true data manifold. SSC-EKE then facilitates the unbiased pairwise constraints to the graph Laplacian from known data labels with high confidence [43]. VIME includes a novel pretext task of estimating mask vectors from corrupted tabular data and a data augmentation step with a pre-trained encoder [44]. VIME has been shown to outperform ElasticNet [45], Context Encoder [46], and MixUp [47] when predicting six different blood cell traits of 300,000 samples using single nucleotide polymorphism (SNPs) information of 1,000 to 100,000 labeled samples [44].

In our supervised analysis, the RF and NN classifiers demonstrated variability in performance when using pseudo-labeled samples. While both models benefited from the additional SS labeled data, the NN had at least 20% higher accuracy in all models compared to the respective RF models. As our models utilized a significantly reduced set of samples compared to the entire training set described by Capper et al. [8], our findings likely reflect the NN classifier's ability to learn with fewer total examples per class even at baseline. Notably, the RF models appeared less sensitive to noisy data in the training set, whereas the NN models showed degradation from baseline with only low-confidence pseudo labels.

Overall, our analysis shows that SSL can be used to label or relabel methylation array samples for clinical diagnostics. In the clinical environment, this approach may be helpful to (1) improve the performance of supervised classifiers by utilizing public methylation data, (2) provide ground truth labels for validation purposes when other traditional approaches appear to be more costly, or (3) modify labels after more granular classification schemes are introduced.

## Methods

### Processing methylation data

All semi-supervised models were trained on genome-wide DNA methylation data from the CNS tumor reference cohort (GSE90496), consisting of 2,801 samples from 75 methylation class families (MCF), which include 91 methylation subclasses (MC) [8]. All methylation data, including those from GSE90496 and GSE109379, were processed in R (http://www.r-project.org, version 4.0.2), using several packages from Bioconductor and other repositories. Specifically, array data were preprocessed using the *minfi* package (v.1.36.0) [48]. Background correction with dye-bias normalization was performed for all samples using noob (normal-exponential out-of-band) with a “single” dye method [49] with preprocessFunNorm. Probe filtering was performed after normalization. Specifically, probes located on sex chromosomes containing a nucleotide polymorphism (dbSNP132 Common) within five base pairs of and including the targeted CpG-site or mapping to multiple sites on hg19 (allowing for one mismatch), and cross-reactive probes were removed from the analysis. After the filtering process, 438,370 probes remained.

### Training and validation of semi-supervised learning (SSL) models

The *ssc* R package (v2.1–0) [50] was used to build and train SSL models. First, the standard deviation for each probe across all 2,801 samples from GSE90496 was calculated. Input features for SSL models were the 5072 probes with a standard deviation greater than 0.3. The train and test data for SSL models are depicted in Fig. 1. Briefly, 30% of DNA methylation data from GSE90496 were kept aside as an inductive testing data set to independently evaluate the performance of each SSL model. The remaining 70% of the data was used as a training set. The training data were proportionally partitioned into a labeled and an unlabeled set. Specifically, 50% of the training data were used as labeled examples, while the remaining 50% (of the 70%) were used as unlabeled examples or as a transductive testing set. The partitioning process was bootstrapped seven times. Each MC or MCF was proportionally sampled for every bootstrap to ensure that the class distributions were similar to the original class distributions.

### Performance metrics

After predicting MC and MCF for both transductive and inductive testing sets, the one-vs-all multiclass performance for each model was evaluated based on the average accuracy, specificity, precision, and recall of all splits. A model with the highest balanced accuracy, specificity, precision, and recall in both testing data sets was selected as the final model for performing threshold analysis and prediction for GSE109379 samples. Balanced accuracy was used to compare performance across models as it is a better judge in the imbalanced class setting, in which some tumor classes are a lot rarer than others and have much smaller sample sizes (< 10 samples in a group) (Additional file 1: Figs. S3 and S4). Balanced accuracy is insensitive to imbalanced class distribution, and it gives more weight to the instances coming from minority classes. On the other hand, accuracy treats all instances alike and usually favors the majority class [51]. First, recall for the algorithm on each class was computed, then the arithmetic mean of these values was calculated to find the final balanced accuracy score. It is "balanced" because each class is represented by its recall; thus, it has the same weight and importance, regardless of its size [52].

### Semi-supervised classifier score calibration

Distributions of the scores generated from the best semi-supervised model (SETRED-SVM) varied between classes, making inter-class comparisons difficult. Furthermore, these scores appeared to spread widely from 0 to 1 when they were used to assign the correct class labels (Fig. 4A), suggesting that they do not reflect well-calibrated class probabilities or certainties of predictions. To have comparable scores among classes and better estimates of the confidence of individual predictions, we performed a calibration process using an L2-penalized, multinomial, logistic regression model described in Capper et al. [8]. The model was fitted with the methylation class as the response variable and the transductive prediction scores as explanatory variables using the *glmnet* (v.4.1–2) R package [53]. The penalization parameter was determined by running tenfold cross-validation, and the λ that gave the minimum mean cross-validated error was chosen.

### Threshold analysis

Finding an optimal cutoff for diagnostic tests is usually necessary to maintain confidence in clinical settings. For example, in brain tumor classification, the cost of false negatives (i.e., a tumor cannot be classified) is usually more tolerable than the cost of false positives (i.e., a tumor is falsely predicted to a methylation class). As such, high specificity (high true negative rate) is preferable. If there are no preferences regarding specificity and sensitivity, the optimal cutoff can be chosen by maximizing the Youden Index (specificity + sensitivity − 1) [38].

In this study, the goal was to demonstrate the utility of SSL in improving a supervised classifier performance; hence, no specific metric was preferred. To find a common cutoff for all MC/MCF, ROC analysis [54] was performed, and the optimal cutoff was chosen by maximizing the Youden Index (balance between specificity and sensitivity). Since ROC analysis is usually designed for binary classification problems, we converted the multiclassification problem here into a binary problem so that a common threshold could be defined for all classes instead of having 91 individual thresholds for 91 methylation classes. Based on the maximal calibrated MC/MCF scores, a binary classification problem was defined as follows: samples that were correctly classified at these scores were considered "classifiable," while samples that were incorrectly classified were considered “non-classifiable”. Under this definition, an optimal threshold (≥ 0.8) was selected, and the area under the curve (AUC) [55], specificity, sensitivity, and precision were evaluated using the *pROC* (v. 1.17.0.1) [56] and *ROCR* (v.1.0–11) [57] R packages.

### Training and testing supervised and deep learning classifiers

A random forest (RF) and a neural net (NN) classifier were built using the python *scikit-learn* [58] and *keras* [59] libraries. The NN was constructed as a sequential model with three dense layers. The first and second layers contained 1000 and 500 units, respectively, with a *he_uniform* kernel initializer and *relu* activation function. The last layer consisted of a softmax layer mapping to either 75 methylation families or 91 subclasses. NN models were fit with a batch size of 6 and 20 epochs. Additional file 1: Fig. S2 showed the four combinations of training and test sets that were used to build eight supervised models and provided a schematic view of the training and testing process for the supervised models. Briefly, 30% of GSE90496 samples (841 samples), representing the inductive test set for the SSL models, were held out as an independent test set for each RF model. The first RF model was trained with the same 35% GSE90496 data (labeled samples used during the training of the best SETRED-SVM classifier). This model is referred to as the baseline model for evaluating SSL pseudo labels. All other RF models were trained using additional SSL labels from the 35% remaining GSE90496 samples plus SSL labels from the GSE109379 samples with or without threshold constraints. Balanced accuracy and weighted recall were computed for each RF after five repeated stratified threefold cross-validation and after predicting the labels of the inductive testing set at *random_state* = 123,456. The 70–30 split was resampled seven times to create seven independent hold-out test sets to better estimate the accuracy and errors of the supervised models. To address the imbalanced problem in the input data set during training, we computed the weight for each class and sample according to its corresponding class weight with the ‘balanced’ parameter in the compute_class_weight and compute_sample_weight functions within *scikit-learn*.

## Supplementary Information

**Additional file 1.** Supplementary figures and legends. Supplementary tables.

## Data Availability

Data were downloaded from the NCBI database with GSE90496 and GSE109379. R and Python codes are uploaded to https://github.com/stjude/Semisupervised_Learning.git and available upon request.

## Abbreviations

AI: Artificial intelligence
AUC: Area under the curve
MC: Methylation subclass
MCF: Methylation class family
NN: Neural net
RF: Random forest
ROC: Receiver and operating characteristic
SETRED-SVM: Self-train with editing method using support vector machine as the base learner
SSL: Semi-supervised learning
