# Supplementary material for: Comprehensive study of semi-supervised learning for DNA methylation-based supervised classification of central nervous system tumors
Source: BMC Bioinformatics. 2022 Jun 8;23:223. doi: 10.1186/s12859-022-04764-1 (PMC9178802; doi:10.1186/s12859-022-04764-1)
Supplement: Supplementary file 1 — Additional file 1. Supplementary figures and legends. Supplementary tables. [file 12859_2022_4764_MOESM1_ESM.pdf]

SUPPLEMENTARY FIGURES AND LEGENDS

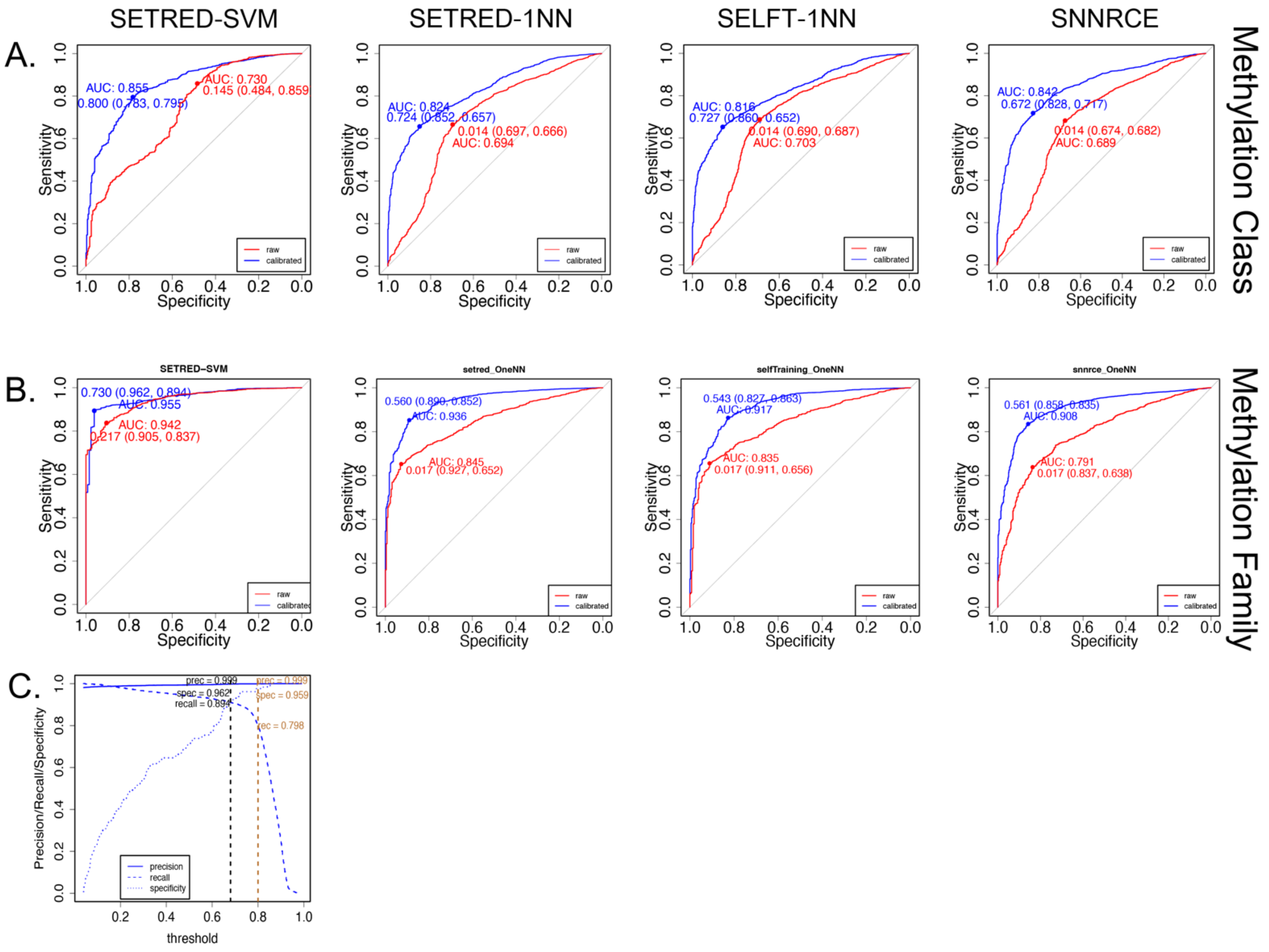

Figure S1. ROC and threshold analysis for raw and calibrated scores of predicting methylation subclass (MC) and family (MCF). ROC analysis of 4 different SSL models using maximal aggregated raw (red) and calibrated (blue) predicted scores with the area under the curve (AUC) and the corresponding Youden Index at which we had a balance between specificity and sensitivity that maximized the Youden Index. (Cin predicting (A) methylation subclasses (B) methylation families. (C) Specificity, sensitivity (recall), and precision at different thresholds using MCF calibrated scores provided by the best SETRED-SVM model. The black vertical line represents the suggested threshold by the threshold analysis. The gold vertical line represents the suggested threshold (≥ 0.8) as seen in predicting methylation subclasses (Figure 5).

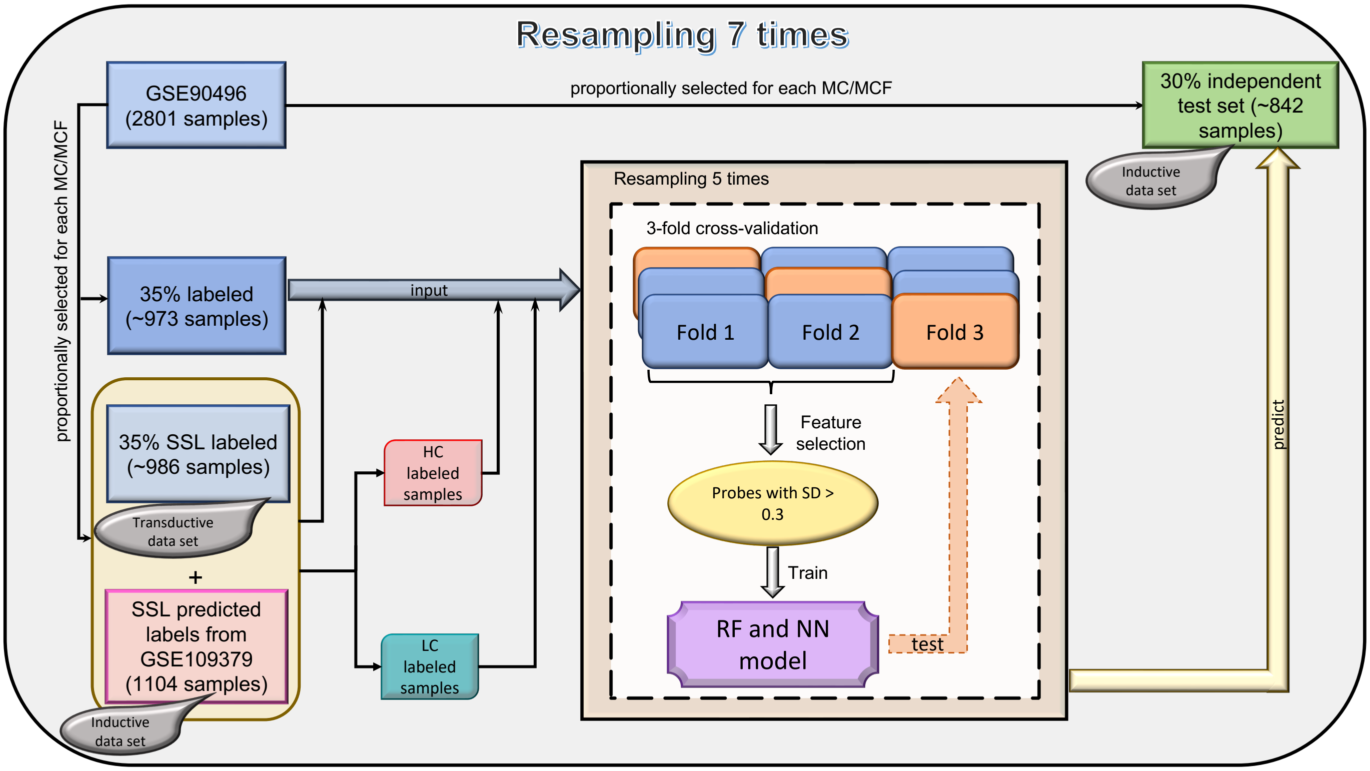

Figure S2. Training and testing scheme for the supervised random forest (RF) and neural network (NN) classifiers. Thirty percent of GSE90496 (842 samples) data was kept as an independent test set for supervised models and referred to as inductive test sets for SSL models. Baseline models were trained with the 35% GSE90496 data (labeled samples used during the training of the best SETRED-SVM classifier). All other models were trained using additional SSL labels from the 35% remaining GSE90496 samples +/- SSL labels from the GSE109379 samples with or without threshold constrain. The GSE109379 data set is collected from a prospective cohort described in [1] consisting of 1,104 samples with given diagnostic categories comprising of 64 different histopathological entities and pediatric cancers. Balanced accuracy and weighted recall were computed for each RF after seven repeated stratified 3-fold cross-validation and after predicting the labels of the inductive testing set. The 70-30 split was resampled seven times to create seven independent holdout test sets to better estimate the supervised classifiers' accuracy and errors. High confidence (HC) labeled samples have calibrated SSL prediction scores of at least 0.8. Low confidence (LC) labeled samples have calibrated SSL prediction scores below 0.8

A.

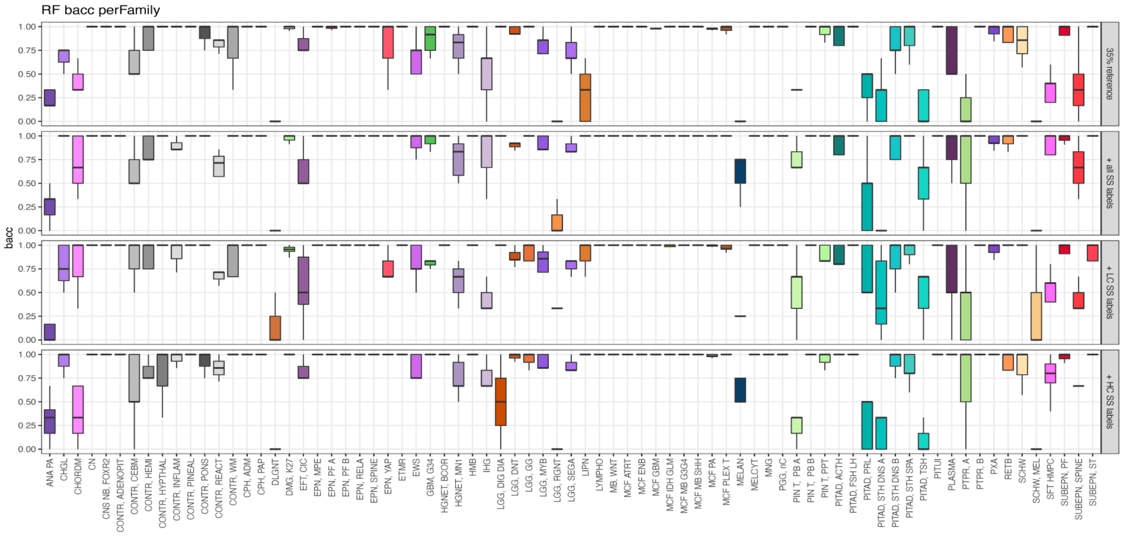

B.

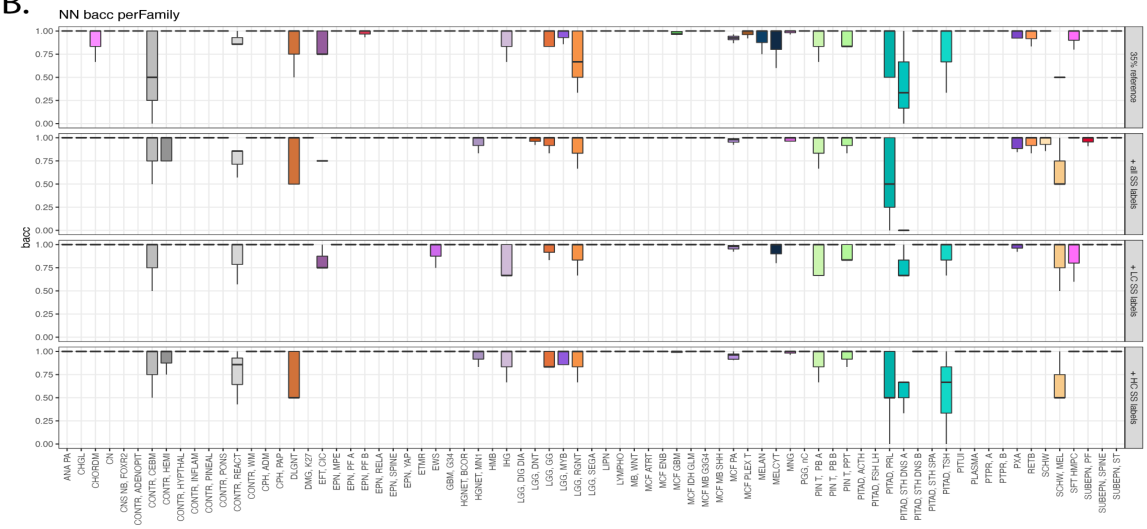

Figure S3. Boxplots representing the average balanced accuracy of random forest (RF) and neural net (NN) models in predicting methylation family for the seven randomly sampled inductive test sets from the GSE90496 reference data. (A) Balanced accuracy of the RF classifiers for each methylation family. (B) Balanced accuracy of the NN classifiers for each methylation family. The first row in each panel shows the performance of the baseline models trained with 35% of the reference data. Models in other rows were trained in addition of semi-supervised learning predicted labels from GSE90496 and GSE109379 as described in Figure S2. Specifically, the second row shows the performance of RF and NN models trained in addition of all semi-supervised learning predicted labels. The third and fourth rows show the performance of RF and NN models that were additionally trained with low- or high-confidence semi-supervised learning labels.

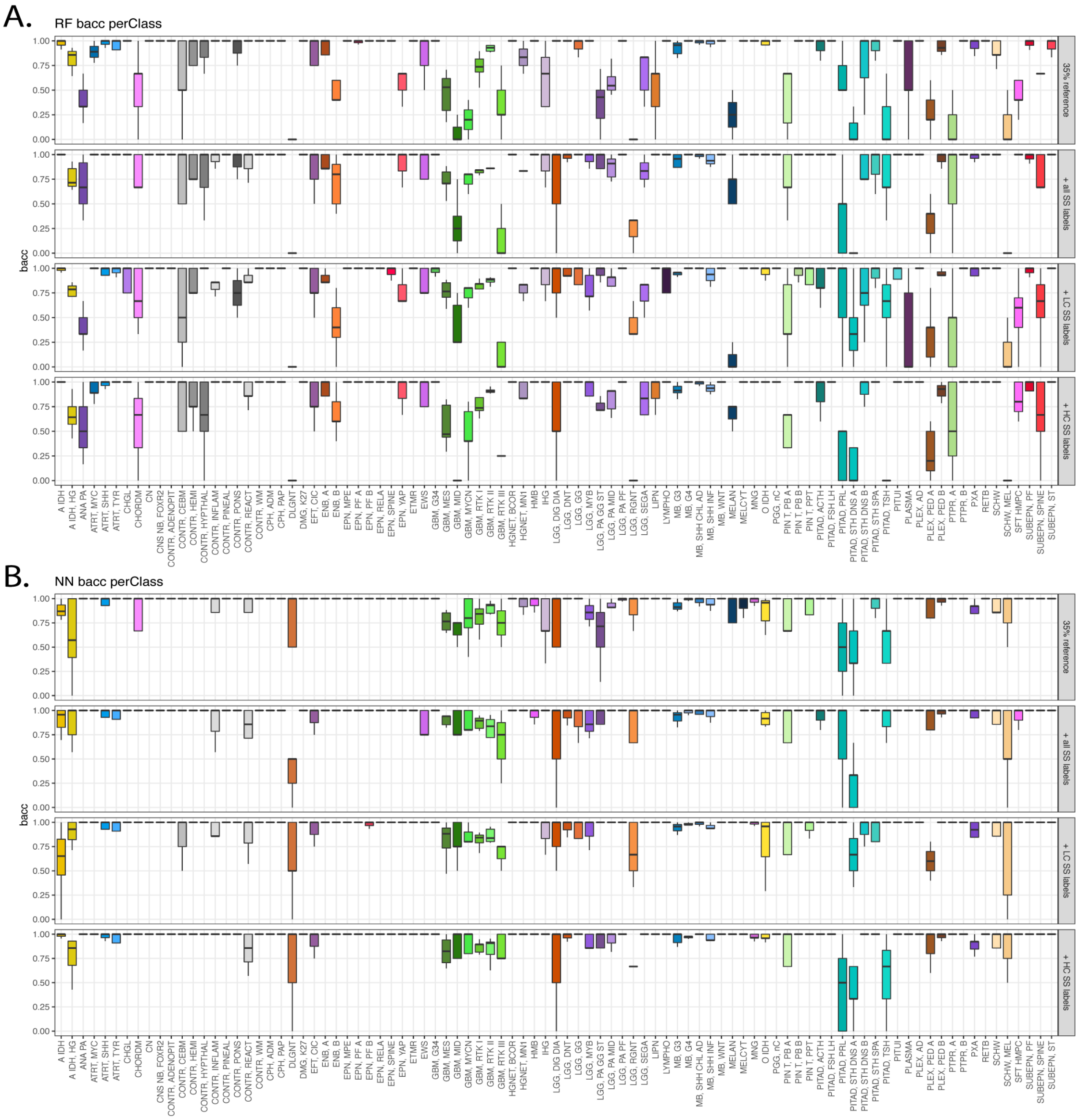

Figure S4. Boxplots representing the average balanced accuracy of random forest (RF) and neural net (NN) models in predicting methylation subclass for the seven randomly sampled inductive test sets from the GSE90496 reference data. (A) Balanced accuracy of the RF classifiers for each methylation subclass. (B) Balanced accuracy of the NN classifiers for each methylation subclass. The first row in each panel shows the performance of the baseline models trained with 35% of GSE90496 labels. Models in other rows were trained in addition of semi-supervised learning predicted labels from GSE90496 and GSE109379 as described in Figure S2. Specifically, the second row shows the performance of RF and NN models trained in addition of all semi-supervised learning predicted labels. The third and fourth rows show the performance of RF and NN models that were additionally trained with either low confidence or high confidence semi-supervised learning labels, respectively.

SUPPLEMENTARY TABLES

Table S1. The 11 semi-supervised learning models being evaluated

Methods	Addition Mechanism	Classifiers	Learning paradigm	Teaching	Base classifier
Self-training [2]	incremental	single	single	self	1-NN
Self-training	incremental	single	single	self	C5.0
Self-training	incremental	single	single	self	SVM
SETRED [3]	amending	single	single	self	1-NN
SETRED	amending	single	single	self	C5.0
SETRED	amending	single	single	self	SVM
SNNRCE [4]	amending	single	single	self	1-NN (fixed)
Tri-training [5]	incremental	multi	single	mutual	1-NN
Tri-training	incremental	multi	single	mutual	C5.0
Tri-training	incremental	multi	single	mutual	SVM
Democratic-Co [6]	incremental	multi	multi	mutual	1-NN/SVM/C5.0

References:

1. Capper D, Jones DTW, Sill M, Hovestadt V, Schrimpf D, Sturm D, Koelsche C, Sahm F, Chavez L, Reuss DE *et al*: **DNA methylation-based classification of central nervous system tumours**. *Nature* 2018, **555**(7697):469-474.
2. Yarowsky D: **Unsupervised word sense disambiguation rivaling supervised methods**. In: *Proceedings of the 33rd annual meeting on Association for Computational Linguistics; Cambridge, Massachusetts*. Association for Computational Linguistics 1995: 189–196.
3. Li M, Zhou Z: **Setred: Self-training with editing**. In: *Advances in Knowledge Discovery and Data Mining*. vol. 3518: Springer Berlin Heidelberg; 2005: 611-621.
4. Wang Y, Xu X, Zhao H, Hua Z: **Semi-supervised learning based on nearest neighbor rule and cut edges**. *Knowledge-Based Systems* 2010, **23**(6):547-554.
5. Zhou Z, Li M: **Tri-training: exploiting unlabeled data using three classifiers**. *IEE* 2005, **17**(Transactions on Knowledge and Data Engineering):1529-1541.
6. Zhou Y, Goldman S: **Democratic co-learning**. *IEEE* 2004(IEEE 16th International Conference on Tools with Artificial Intelligence (ICTAI)):8.
